# Carbapenemase Production and Detection of Colistin-Resistant Genes in Clinical Isolates of *Escherichia Coli* from the Ho Teaching Hospital, Ghana

**DOI:** 10.1155/2022/1544624

**Published:** 2022-06-27

**Authors:** John Gameli Deku, Kwabena Obeng Duedu, Godsway Edem Kpene, Silas Kinanyok, Patrick Kwame Feglo

**Affiliations:** ^1^Department of Medical Laboratory Sciences, School of Allied Health Sciences, University of Health and Allied Sciences, Ho, Ghana; ^2^Department of Biomedical Science, School of Basic and Biomedical Sciences, University of Health and Allied Sciences, Ho, Ghana; ^3^Department of Clinical Microbiology, School of Medicine and Dentistry, KNUST, Kumasi, Ghana

## Abstract

**Background:**

Effective and successful treatment of infectious diseases is a significant gain in clinical settings. However, resistance to antibiotics, especially the last-resort medicines, including carbapenems and colistin is on the rise.

**Aim:**

The aim of this study was to detect carbapenemase production and colistin-resistant genes in clinical isolates of *Escherichia coli. Method*. The study was a cross-sectional study carried out from July 2018 to June 2019. One hundred and thirty-five nonrepetitive *E. coli* isolates obtained from various clinical samples were screened for carbapenemase production using meropenem (10 *μ*g) and imipenem (10 *μ*g) disks. Screened-positive isolates were further subjected to a confirmatory test using modified carbapenem inhibition method (mCIM). Deoxyribonucleic acid (DNA) was extracted from all the isolates to detect colistin-resistant genes by polymerase chain reaction. Data were analyzed using GraphPad Prism version 8.00 for Windows and IBM SPSS version 26 (IMB Corp. New York, USA).

**Results:**

Of the 135 isolates, 2 were screened positive for carbapenemase production but tested negative to mCIM. With the colistin-resistant genes, only *mcr-1* and *mcr-*2_700bp were detected in 3 of the *E. coli* isolates, representing 2.2%. The *mcr-1* was detected in a high vaginal swab sample of a female aged between 65 and 84 years. *Mcr-*2_700bp was also detected in urine and blood samples of the patients.

**Conclusion:**

The study investigated the presence of carbapenemase and colistin-resistant genes in *E. coli* organisms. The absence of carbapenemase in the isolates and the detection of colistin-genes call for strict infection prevention and control practices to prevent their introduction and spread to other bacterial species, respectively.

## 1. Introduction

The discovery of antimicrobial agents has played a significant role in reducing the incidence of illness and death caused by microbial agents. The successful treatment of infectious diseases is one of the remarkable gains in modern medicine. The expansion in health systems and resources, in addition to improved production technologies across the globe, has also led to an increase in availability and ease of access to antimicrobials, especially, over-the-counter drugs. However, the improved access to these antimicrobials accompanied by poor practices has contributed to bacteria, including *E. coli*, to develop resistance to these life-saving drugs.

The rising rates of antimicrobial resistance continue to pose a grave threat to human health globally but more especially, in low- and middle-income countries, partly due to the high burden of communicable diseases [1]. It is estimated that antimicrobial resistance will cause 10 million deaths globally and an economic loss of over 100 trillion US dollars by the year 2050 [2]. The problem of antibiotic resistance is compounded by the resistance of the bacterial organisms to last-resort antibiotics, including carbapenems and colistin.

Until recently, carbapenems were the choice of treatment for multidrug-resistant Gram-negative bacterial infections [3]. Despite being generally considered as last-resort antibiotics [4], resistance to this class of antibiotics has been documented in previous studies [5–7]. Resistance of bacteria to carbapenems is due to the production of carbapenem-hydrolyzing enzymes called carbapenemases. These bacteria have the ability to spread rapidly within the hospital environment and from one country to another [8]. The occurrence of an outer membrane porin deficiency and the expression of a plasmid-mediated class C-lactamase have been reported to be responsible for carbapenem resistance in *E. coli* [9].

Colistin, on the other hand, has been used in treating infections caused by bacterial species in farm animals. The main indications for colistin use in veterinary settings are the prevention and treatment of infections caused by *Enterobacteriaceae* but it has also been used as a growth enhancer in terrestrial and aquatic animals [10, 11]. In recent times, colistin is being used in humans as a last-resort antibiotic in the treatment of infections caused by carbapenem-resistant *Enterobacteriaceae*. This has prompted more accurate and careful monitoring of resistance to this polypeptide [12]. The aim of this study was to investigate the production of carbapenemase and colistin-resistant genes in clinical isolates of *E. coli* from the Ho Teaching Hospital, Ghana.

## 2. Materials and Methods

### 2.1. Study Design and Study Area

The study was a cross-sectional study carried out at the Ho Teaching Hospital from July 2018 to June 2019. The hospital is a 241-bed capacity tertiary medical facility located in the regional capital of the Volta Region, Ho. The microbiology department of the hospital receives laboratory requests for various microbiological analyses from various units and departments of the hospital, as well as from the entire region and neighboring health facilities. Analysis of the samples were conducted in the Duedu Laboratory in the Department of Biomedical Science, School of Basic and Biomedical Sciences of the University of Health and Allied Sciences, in Ho which studies microbial ecology and biotechnology.

### 2.2. The Bacterial Isolates

*E. coli* isolates were cultured from various clinical specimens on MacConkey agar (Oxoid, UK) and blood agar (Oxoid, UK). The plates were incubated aerobically for 24 hours at 37°C. Growths suspected to be *E. coli* were confirmed using Gram stain reaction, triple sugar fermentation test, citrate test, urease test, indole test, Voges–Proskauer, and methyl red tests. Confirmation was done by detection of the presence of *uidA* and *uspA* genes that are specific to *E. coli* by PCR. Primers used to amplify these genes are shown in Table 1. The organisms isolated and confirmed as *E. coli* were stored in glycerol stocks in a −80°C freezer and later used for other tests. Quality control organisms used in the study were *E. coli* ATCC 25922 and *Klebsiella pneumoniae* NCTC 13442. *K. pneumoniae* was used as a negative control for the biochemical tests except for triple sugar ion fermentation in which both *E. coli* and *K. pneumoniae* share common characteristics.

### 2.3. Revival of Isolates and DNA Extraction

The stored *E. coli* isolates were retrieved from the freezer, and the surface was scraped aseptically and emulsified in a 30 ml Luria Bertani broth (Oxoid, UK) and incubated overnight in a shaking incubator. The genomic DNA was extracted according to a previous technique used by Deku, Duedu [14].

### 2.4. Screening of *E. coli* Isolates for Carbapenemase Production

The disk diffusion Kirby-Bauer antimicrobial susceptibility testing method was used to detect carbapenemase production against meropenem (10 *μ*g) and imipenem (10 *μ*g). Three well-isolated colonies of *E. coli* were touched and emulsified in 4 ml of a sterile phosphate buffered saline. The density of the inoculum was adjusted to 0.5 McFarland. The Mueller–Hinton agar (Oxoid, UK) was inoculated by dipping a sterile swab into the inoculum and streaking the entire surface of the medium. A duration of ten minutes was allowed to enable the surface moisture to dry before the antibiotic disks were placed on the medium using sterile forceps. The inoculated plates containing the antibiotic disks were incubated aerobically at 37°C for 16–18 hours. The diameter of the zone of inhibition was measured using a ruler and was recorded. Isolates that were resistant (zone diameter ≤19 mm) to either one or two of the carbapenem antibiotic disks were suspected of carbapenemase production and were subjected to modified the carbapenem inhibition method (mCIM).

### 2.5. Confirmation of Carbapenemase Production Using mCIM

This test was performed by emulsifying a 10 *μ*l loopful of *E. coli* culture from the Mueller–Hinton agar into 400 *μ*l of sterile saline [15]. A 10 *μ*g meropenem disk was completely immersed into the suspension using sterile forceps. The inoculum containing the immersed disk was incubated for four hours at 37°C aerobically. Immediately before the completion of the saline meropenem incubation, a 0.5 McFarland suspension was prepared using a controlled *E. coli* ATCC 25922 organism in sterile normal saline. The Mueller–Hinton agar was inoculated with the control organism according to a routine disk diffusion procedure and the plates were allowed to dry for 10 minutes [16]. The meropenem disk was removed from the suspension using a loop. Excess fluid was removed from the disk by dragging and pressing the loop along the inside wall of the tube. The disk was added to the seeded Mueller–Hinton plate and incubated at 37°C for 18 hours aerobically. If the isolate was a carbapenemase producer, the meropenem in the susceptibility disk would have been inactivated by allowing uninhibited growth (6 mm–15 mm) of the susceptible *E. coli* strain (positive carbapenemase test). Disks incubated in suspensions that did not contain carbapenemase produced a clear inhibition zone (≥19 mm) (negative carbapenemase test) [16, 17]. A zone size of 16 mm–18 mm was considered as indeterminate [16].

### 2.6. Molecular Detection of Colistin-Resistant Genes

The DNA extracted from the 135 isolates were subjected to polymerase chain reaction to determine the amplification of *mcr* genes using primers given in Table 2. The amplification was done using one taq quick-load 2x master mix with standard buffer. A total of 12.5 *μ*l reaction volume was used, comprising 6.25 *μ*l of one taq quick-load 2x master mix with standard buffer, 0.25 *μ*l each of 10 *μ*M forward and reverse primers, 1 *μ*l of template DNA, and 4.75 *μ*l of nuclease-free water. In all cases, the running conditions were as follows: 1 cycle of initial denaturation at 94°C for 10 minutes, followed by 30 cycles of denaturation at 94°C for 30 seconds, annealing (at temperature specific for primers) for 60 seconds, and extension at 72°C for 60 sec/kb. The final extension was done at 72°C for 10 minutes and held at 4°C. Thermal cycling was done using a thermocycler (Eppendorf, USA).

### 2.7. Statistical Analysis

GraphPad Prism version 8.00 for Windows and IBM statistical software Package for the Social Sciences (SPSS Inc. Chicago, USA) version 26.00 were used for the statistical analysis. Data obtained from the laboratory analysis were verified and exported to the statistical software for analysis. Data analysis included descriptive statistics, bivariate, and multivariate analyses by performing cross tabulations to obtain Fisher's chi squares for determination of statistical dependencies and risk associations of study variables.

### 2.8. Ethical Consideration

Ethical approval was granted by a joint committee of Komfo Anokye Teaching Hospital and School of Medical Sciences and Dentistry, Kwame Nkrumah University of Science and Technology, (with reference No. CHRPE/AP/204/18) before the commencement of the study. Data on study participants were anonymous and nonlinked to any of the participants. Isolates from the study participants were assigned study identification numbers.

## 3. Results

The study characterized a total of 135 clinical *E. coli* isolates recovered from the various clinical specimens of patients who visited the Ho Teaching Hospital for healthcare. Majority of the organisms (82.2%) were isolated from female participants and were mostly obtained from the 25–44 years age group 60 (44.4%). Christianity 128 (94.8%) was identified as the most professed religion among the patients. The isolates were mostly recovered from outpatients 98 (72.6%), who presented with urinary tract infections. Details of these results are presented in Table 3.

When the 135 isolates were screened for carbapenemase production, only 2 were resistant to meropenem and imipenem. The suspected carbapenemase-producing isolates were however negative in the modified carbapenem inactivation method to confirm the production of carbapenemase.

With the colistin-resistant genes, it was observed that only resistant genes *mcr-1* and *mcr-2_700bp* were detected in 3 of the *E. coli* isolates, representing 2.2% prevalence. The *mcr-1* was detected in a high vaginal swab sample of a female aged between 65–84 years, whereas *mcr-*2_700 *bp* was also detected in both urine and blood samples of the patients, aged <5 years and between 5 and 24 years. See Table 4.

## 4. Discussion

*E. coli* is a member of normal microflora of gastrointestinal tract of man and other animals and belongs to the family of *Enterobacteriaceae*. Although most strains are nonpathogenic in the gut, few can cause disease in man in the intestine or outside the intestine. Infections caused by this organism are treated with cephalosporins. However, there are reports of resistance to this class of antimicrobials due to the production of extend-spectrum beta-lactamases (ESBL). This has necessitated the use of carbapenem drugs. Carbapenems are antimicrobial agents that are used as last resorts in the treatment of infections caused by ESBL-producing bacteria.

There is, however, a growing concern about the emergence and spread of carbapenemase-producing bacteria. Contrary to the global increase in carbapenemase-producing organisms [21–23], our study detected two meropenem- and imipenem-resistant *E. coli* isolates that tested negative to mCIM. The screened-positive but negative confirmatory test may not connote carbapenemase production as other factors such as increased efflux pump activity and porin loss can also cause resistance [24, 25]. For infection prevention and control purposes, it is important to draw the distinction between carbapenem resistance due to carbapenemase production and resistance mediated by other mechanisms [15]. This is because most carbapenemase are plasmid encoded and can readily spread form one organism to the other. Since resistant genotypes can be transferred through crossborder migration, there is the need to put in place or enforce the existing infection prevention and control practices in the study area since carbapenemase production has been detected in other African countries, including 2.8% in Morocco [26], 28.6% in Uganda [23], 33.5% in Nigeria [27], and 35.24% in Tanzania [22].

Due to the emergence and rise of carbapenemase-producing bacteria in other countries, there is a need for proactive and adequate preventive measures locally, regionally, and nationally to contain the spread of these resistant bacteria [28]. Although, their detection can be difficult as their presence do not always produce resistant phenotypes on conventional disc diffusion or automated susceptibility testing methods [29], early detection through targeted laboratory protocols and containment of spread through comprehensive infection control measures are among appropriate preventive measures to take [28].

In this study, 3 *E. coli* isolates harbored one colistin resistant gene each, representing 2.2% of the total isolates. The resistance proportion recorded in this study is alarming since colistin has been regarded as a last-resort antibiotic [30, 31]. Previous studies recorded a lower prevalence in comparison with our current study [32–35]. These studies were designed to detect only *mcr-1* resistant genotype, contrary to the current study where various oligonucleotide primers were used to detect a wide range of colistin resistant genes, including *mcr-2, mc3-3,* and *mcr-4*, among others. Other studies have reported a higher prevalence in comparison with this current study in Nigeria 8.3% [30] and Thailand 4.7% [36].

As at the year 2020, thirty-six studies were conducted to detect the presence of colistin-resistant genes in Africa and twenty-seven of these studies were carried out in North African countries [37]. The *mcr* genes have been reported in only six of the African countries and the distribution of these genes is also changing [37]. In those studies, *mcr-1, mcr-2, mcr-3*, and *mcr-4* genes were detected in Northern Africa, while only *mcr-1* genotypes were detected in Southern and Central African countries. Only two studies were conducted in Western Africa, specifically in Nigeria where only the *mcr-1* genotype was detected. Our current study recorded *mcr-1* 1 (0.7%) and *mcr-2*–700 bp 2 (1.5%) among the 135 isolates tested.

Colistin has not been in use in the study hospital. It is possible that the 3 isolates carrying the colistin-resistant genes were present in the community where this class of antibiotic is used for agricultural purposes, rather than arising as a result of selective pressure in the hospital [38]. This calls for a judicious use of this antimicrobial agent in animals so as to retain its efficacious activity.

The predominant colistin-resistant genotype detected in this study was *mcr-*2_700 bp, with *mcr-1* being the least. The *mcr-2* predominance was in contrast with previous studies which reported *mcr-1* majority [38]. A study conducted in 36 countries across the globe reported the detection of plasmid-mediated *mcr-1*, indicating the widespread presence of this colistin-resistant genotype [39]. The detection of other colistin-resistant genes in this study calls for regular monitoring and surveillance to prevent the spread of these resistant genotypes.

## 5. Conclusion

In conclusion, the study investigated the presence of carbapenemase and colistin-resistant genes in the *E. coli* organism. The absence of carbapenemase in the isolates coupled with the presence of *mcr* genes calls for strict infection prevention and control practices to prevent their introduction and spread to other bacterial species, respectively.

## Figures and Tables

**Table 1:** Oligonucleotide primers used for the molecular identification of *E. coli.*

Target gene	Sequence (5′-3′)	Amplicon size (bp)	Reference
*uspA*	F_CCGATACGCTGCCAATCAGT	884	[13]
	R_ACGCAGACCGTAGGCCAGAT		

*uidA*	F_CTGGTATCAGCGCGAAGTCT	556	[13]
	R_AGCGGGTAGATATCACACTC		

**Table 2:** Primers for detection of colistin-resistant genes.

Target gene	Primer	Sequence (5′-3′)	Amplification product (bp)	Annealing temperature (°C)	Reference
*mcr*-1	*mcr*-1F	ATCAGCCAAACCTATCCCATCG	1257	55	[18]
	*mcr*-1R	GCAGACGCACAGCAATGCCTAT			

*mcr*1_320 bp	*mcr*1_320 bp_F	AGTCCGTTTGTTCTTGTGGC	320	58	[19]
	*mcr*1_320 bp_R	AGATCCTTGGTCTCGGCTTG			

*mcr*-2	*mcr*-2F	GCGATGGCGGTCTATCCTGTAT	378	55	[18]
	*mcr*-2R	TGCGATGACATGGGGTGTCAGC			

*mcr*-2_700 bp	*mcr*2_700 bp_F	CAAGTGTGTTGGTCGCAGTT	715	58	[19]
	*mcr*2_700 bp_R	TCTAGCCCGACAAGCATACC			

*mcr*-3	*mcr*-3F	TATGGGTTACTATTGCTGG	814	55	[18]
	*mcr*-3R	CTACCCTGATGCTCATCG			

*mcr*3_900 bp	*mcr*3_900 bp_F	AAATAAAAATTGTTACGCTTATG	929	58	[19]
	*mcr*3_900 bp_R	AATGGAGATCCCCGTTTTT			

*mcr*-4	*mcr*-4F	GTCATAGTGGTATAAAAGTACAG	669	55	[18]
	*mcr*-4R	CCACCGTCTATCAGAGCCAAC			

*mcr*_4_1100 bp	*mcr*_4_1100 bp_F	TCACTTTCATCACTGCGTTG	1116	58	[19]
	*mcr*_4_1100 bp_R	TTGGTCCATGACTACCAATG			

*mcr*-5	*mcr*5_F	ATGCGGTTGTCTGCATTTATC	1644	58	[20]
	*mcr*5_R	TCATTGTGGTTGTCCTTTTCTG			

m*mcr*-5	*mcr*-5F	GCGGTTGTCTGCATTTATCAC	1042	50	[18]
	*mcr*-5R	CTTTGAAAACCTGTCTTTGGCA			

*mcr*-6	*mcr*-6F	GTCCGGTCAATCCCTATCTGT	556	55	[18]
	*mcr*-6R	ATCACGGGATTGACATAGCTAC			

*mcr*-7	*mcr*-7F	TGCTCAAGCCCTTCTTTTCGT	892	55	[18]
	m*mcr*-7R	TTCATCTGCGCCACCTCGT			

*mcr*-8	*mcr*-8F	AACCGCCAGAGCACAGAATT	667	60	[18]
	*mcr*-8R	TTCCCCCAGCGATTCTCCAT			

**Table 3:** Sociodemographic characteristics of study respondents.

Parameter	Frequency	Percentage
*Age category (years)*		
<5	9	6.7
5–24	14	10.4
25–44	60	44.4
45–64	31	23
65–84	21	15.6

*Gender*		
Male	24	17.8
Female	111	82.2

*Religion*		
None	5	3.7
Christian	128	94.8
Islam	2	1.5

*Specimen type*		
Urine	98	72.6
Nonurine	37	27.4

*Patient status*		
Outpatient	98	72.6
Inpatient	37	27.4

**Table 4:** Proportional distribution of colistin-resistant genes among study participants.

Parameter	Total	*mcr* 1	*mcr* 1_320 bp	*mcr* 2	*mcr* 2_700 bp	*mcr* 3	*mcr* 3_900 bp	*mcr* 4	*mcr* 4_1100 bp	*mcr* 5–1042	*mcr* 5–1644	*mcr* 6	*mcr* 7	*mcr* 8
*Gender*														
Male	24 (100.0)	0 (0.0)	0 (0.0)	0 (0.0)	0 (0.0)	0 (0.0)	0 (0.0)	0 (0.0)	0 (0.0)	0 (0.0)	0 (0.0)	0 (0.0)	0 (0.0)	0 (0.0)
Female	111 (100.0)	1 (0.9)	0 (0.0)	0 (0.0)	2 (1.8)	0 (0.0)	0 (0.0)	0 (0.0)	0 (0.0)	0 (0.0)	0 (0.0)	0 (0.0)	0 (0.0)	0 (0.0)
Total 1(0.7)		135 (100)	**0 (0.0)**	**0 (0.0)**	**2 (1.5)**	**0 (0.0)**	**0 (0.0)**	(0.0)	**0 (0.0)**	**0 (0.0)**	**0 (0.0)**	**0 (0.0)**	**0 (0.0)**	**0 (0.0)**

*Age category(years)*														
<5	9 (100.0)	0 (0.0)	0 (0.0)	0 (0.0)	1 (11.1)	0 (0.0)	0 (0.0)	0 (0.0)	0 (0.0)	0 (0.0)	0 (0.0)	0 (0.0)	0 (0.0)	0 (0.0)
5–24	14 (100.0)	0 (0.0)	0 (0.0)	0 (0.0)	1 (7.1)	0 (0.0)	0 (0.0)	0 (0.0)	0 (0.0)	0 (0.0)	0 (0.0)	0 (0.0)	0 (0.0)	0 (0.0)
25–44	60 (100.00)	0 (0.0)	0 (0.0)	0 (0.0)	0 (0.0)	0 (0.0)	0 (0.0)	0 (0.0)	0 (0.0)	0 (0.0)	0 (0.0)	0 (0.0)	0 (0.0)	0 (0.0)
45–64	31 (100.0)	0 (0.0)	0 (0.0)	0 (0.0)	0 (0.0)	0 (0.0)	0 (0.0)	0 (0.0)	0 (0.0)	0 (0.0)	0 (0.0)	0 (0.0)	0 (0.0)	0 (0.0)
65–84	21 (100.0)	1 (4.8)	0 (0.0)	0 (0.0)	0 (0.0)	0 (0.0)	0 (0.0)	0 (0.0)	0 (0.0)	0 (0.0)	0 (0.0)	0 (0.0)	0 (0.0)	0 (0.0)

*Specimen type*														
Urine	98 (100.0)	0 (0.0)	0 (0.0)	0 (0.0)	1 (1.0)	0 (0.0)	0 (0.0)	0 (0.0)	0 (0.0)	0 (0.0)	0 (0.0)	0 (0.0)	0 (0.0)	0 (0.0)
Blood	5 (100.0)	0 (0.0)	0 (0.0)	0 (0.0)	1 (20.0)	0 (0.0)	0 (0.0)	0 (0.0)	0 (0.0)	0 (0.0)	0 (0.0)	0 (0.0)	0 (0.0)	0 (0.0)
Ear swab	5 (100.0)	0 (0.0)	0 (0.0)	0 (0.0)	0 (0.0)	0 (0.0)	0 (0.0)	0 (0.0)	0 (0.0)	0 (0.0)	0 (0.0)	0 (0.0)	0 (0.0)	0 (0.0)
HVS	10 (100.0)	1 (10.0)	0 (0.0)	0 (0.0)	0 (0.0)	0 (0.0)	0 (0.0)	0 (0.0)	0 (0.0)	0 (0.0)	0 (0.0)	0 (0.0)	0 (0.0)	0 (0.0)
Pleural	1 (100.0)	0 (0.0)	0 (0.0)	0 (0.0)	0 (0.0)	0 (0.0)	0 (0.0)	0 (0.0)	0 (0.0)	0 (0.0)	0 (0.0)	0 (0.0)	0 (0.0)	0 (0.0)
Sputum	2 (100.0)	0 (0.0)	0 (0.0)	0 (0.0)	0 (0.0)	0 (0.0)	0 (0.0)	0 (0.0)	0 (0.0)	0 (0.0)	0 (0.0)	0 (0.0)	0 (0.0)	0 (0.0)
Wound	14 (100.0)	0 (0.0)	0 (0.0)	0 (0.0)	0 (0.0)	0 (0.0)	0 (0.0)	0 (0.0)	0 (0.0)	0 (0.0)	0 (0.0)	0 (0.0)	0 (0.0)	0 (0.0)

## Data Availability

Data are obtainable from the corresponding author upon satisfactory request.
